# Improving Outcomes in Nosocomial Pneumonia: Recent Evidence and More Challenges

**DOI:** 10.3390/pathogens13060495

**Published:** 2024-06-10

**Authors:** Ihab H. Masri, Bethany Busack, Andrew F. Shorr

**Affiliations:** 1 Pulmonary and Critical Care Medicine, Medstar Washington Hospital Center, Washington, DC 20010, USA; masriehab@gmail.com; 2 Critical Care Medicine, University of Michigan-West, Wyoming, MI 49519, USA; bethany.busack@gmail.com

**Keywords:** antibiotic, diagnosis, mechanical ventilation, microbiology, pneumonia, prevention, resistance

## Abstract

Nosocomial pneumonia (NP) represents a leading nosocomial infection and results in substantial morbidity and cost. Over the last several years, the evidence has evolved which directs our approach to NP. Specifically, the definition of NP and classification of its various subtypes has expanded to capture nuances among various phenotypes of this syndrome. For example, segregating those with hospital-acquired pneumonia (HAP) based on whether they subsequently require mechanical ventilation has been shown to be important. Likewise, newer data indicate the true economic cost of NP and underscore the diverse range of pathogens that can cause NP. Moreover, multidrug-resistant (MDR) bacteria have become a major threat in NP. Fortunately, newer simple preventive strategies have been tested and found to be effective at reducing the incidence of NP. Should prevention fail, a range of new antibiotics have been formally studied in NP and found to be effective. Some of these novel agents have relatively broad ranges of activity and are in vitro active against select MDR organisms. Others, however, are narrower in spectrum and directed against specific problem bacteria. In short, the literature in the field of NP has progressed rapidly, and clinicians require a clear appreciation of these changes so as to improve patient outcomes.

## 1. Introduction

Nosocomial pneumonia (NP) represents a leading potential complication of hospitalization. Although it is not the common nosocomial infection, NP presents a major challenge for both patients and clinicians. Unlike the leading infectious nosocomial process, urinary tract infection, NP remains a diagnostic challenge and can lead to severe excess morbidity and costs [1,2]. Furthermore, NP can present as an indolent process or lead to septic shock. Similarly, NP affects both medical and surgical patients. As such, the rates of NP in its two general forms, hospital-acquired pneumonia (HAP) and ventilator-associated pneumonia (VAP), serve as a key metric in assessing a hospital’s overall quality and safety. Although the Centers for Medicaid and Medicare Services (CMS) in the United States (US) do not expressly address NP in its assessment of hospital quality, the CMS value-based purchasing plan designed to limit hospital-acquired conditions penalizes hospitals for events that may arise as a consequence of NP—such as post-operative respiratory failure and sepsis [3].

The continuing surge in antimicrobial resistance (AMR) additionally complicates NP treatment. A challenge both in Gram-positive and Gram-negative bacteria, multidrug resistant (MDR) pathogens often represent the leading causes of NP. Conversely, antibiotic overuse and abuse in NP certainly contributes to and continues to fuel further AMR. In essence, any physician treating NP faces a major dilemma. On the one hand, it is crucial to ensure that a patient with NP receives initially appropriate antimicrobial therapy. Multiple analyses document that initially inappropriate therapy, defined as either delayed or in vitro inactive antibiotic treatment, increases a patient’s risk for death significantly [4,5]. On the other hand, the clinician must adhere to principles of antibiotic stewardship by aiming not to overtreat NP by either relying on agents that are overly broad in their spectrum or by treating for an excessive duration.

Fortunately, multiple recent trials and reports have examined several central aspects of NP care. Specifically, these analyses have addressed not only the epidemiology and microbiology of NP but have also explored new ways to categorize and to diagnose NP. In addition, there have been major advancements in paradigms for preventing NP. Newer antimicrobial agents for MDR pathogens, moreover, have been tested and are now available to clinicians. Important subtleties distinguish these newer options for NP treatment. Hence, one requires an appreciation of the nuances amongst these various agents. Likewise, a comprehensive and revitalized approach to NP necessitates not only an understanding of the recent trials related to NP treatment but also necessitates a careful evaluation of the newer literature discussing epidemiology, microbiology, and prevention as well. In sum, we present a synthesis of the recent literature on this topic that is important for both intensivists and infectious disease physicians to comprehend. Moreover, we highlight recent studies that we qualitatively believe are important to appreciate if one hopes to apply the most substantial recent evidence to clinical practice.

## 2. Materials and Methods

We conducted a review of recent trials and reports relating to various aspects of NP care. We attempted to limit our search to articles published over the last 5 years. We further sought to identify well-done and high-quality studies in this field by mainly focusing our article selection on either large retrospective analyses or prospective, observational studies and randomized trials. In addition, we sought to be broad in our scope by not limiting our efforts to one aspect of NP. Rather we attempted to be comprehensive in our effort by exploring a range of aspects related to NP.

## 3. Epidemiology

Traditionally, NP has been classified into two broad categories: HAP and VAP. VAP historically has represented a new pneumonia that arises after 24–48 h of mechanical ventilation (MV). Readers should note that VAP is traditionally identified based on a combination of clinical signs and symptoms along with the presence of a new or evolving infiltrate on some form of chest imaging. This is distinct from the concept of the ventilator-associated event (VAE). VAEs, in contrast to VAP, can be either infectious or non-infectious in origin. The concept of the VAE is meant to capture multiple types of complications, ranging from barotrauma to tracheitis. VAEs, like VAP, can lead to morbidity and mortality and thus are emphasized as another metric reflecting the quality of care of ventilated subjects. In other words, VAP is a form of VAE, but not all VAEs represent VAP. Thus, it is important to realize, again, that we are focusing on VAP.

The remaining non-VAP NP was considered to be HAP. Recent evidence, though, suggests that it is important to separate HAP further. More specifically, studies indicate that some patients with HAP who subsequently require MV (vHAP) face distinct outcomes from those who are diagnosed with HAP but who never require invasive ventilatory support (nvHAP). For example, in a recent large retrospective cohort analysis, nvHAP incidence arose in 0.55 out of every 100 hospitalizations. This syndrome was associated with a crude inpatient mortality rate of 22%. Furthermore, nvHAP accounted for up to 7 percent of all hospital deaths [6].

### 3.1. Ventilated Hospital-Acquired Pneumonia 

vHAP, alternatively, more resembles VAP with respect to its impact on resource use and mortality. An analysis of a large administrative database of US patients revealed that vHAP occurs as often as nvHAP—indicating that nearly 50% of patients diagnosed with HAP will progress to require MV. Demonstrating the high severity of illness associated with vHAP, those with vHAP more often met criteria for septic shock than persons with traditional VAP. More importantly, crude hospital mortality in vHAP was higher than that seen among persons suffering from VAP (30% vs. 21%). Similarly, the median length of stay and costs for care were nearly identical for both vHAP and VAP. These similarities reveal that vHAP clearly has a different trajectory and set of outcomes than nvHAP. As such, clinicians recognize the distinctions between vHAP and nvHAP and react accordingly. In other words, patients ventilated for an episode of HAP merit special attention and efforts to insure timely diagnostic and therapeutic interventions. It is crucial to note that the bacterial organisms isolated in vHAP and VAP are similar (see below). This observation underscores that the burden of vHAP is not driven by some particular set of multidrug-resistant (MDR) organisms disproportionately seen in vHAP [7]. Furthermore, differences in crude mortality do not appear to be related to rates of initially inappropriate antibiotic therapy. For example, Zilberberg et al. documented that rates of inappropriate therapy were highest among patients with nvHAP relative to those suffering from other forms of NP [8]. 

### 3.2. Ventilator-Associated Pneumonia 

With respect to VAP, the prevalence of this condition varies across the globe and differs because of factors including but not limited to ICU type, country, and microbiological sampling methodologies. For example, the prevalence of VAP is reportedly higher in middle- and lower-income countries [9]. Also, patients with comorbidities such as trauma, chronic obstructive pulmonary disease (COPD), and various forms of immunosuppression face a higher incidence of VAP. Specifically, trauma patients may have a higher risk of aspiration as a result of their injury. Consequently, in one series of 511 patients, the incidence of VAP reached 17.8% [10]. In a meta-analysis examining patients with traumatic brain injury (TBI), the pooled incidence of VAP exceeded 35% [11]. Conversely, a large cohort study exploring variables associated with VAP revealed that subjects intubated for COPD disproportionately were diagnosed with VAP, even when controlling for the duration of MV [12]. However, with the adoption of multiple preventive strategies, the incidence of VAP has fallen. Dudek et al. concluded that rates of VAP fell by nearly 65% between 2006 and 2012 in several ICUs that they evaluated [13].

For some time, controversy has existed regarding the attributable mortality related to VAP. Based on observational cohort studies completed over two decades ago [14], the attributable mortality rate was believed to exceed 25%. However, in a systemic review of studies reporting mortality for VAP, researchers determined that the attributable mortality associated with VAP could not be quantified due to the heterogenous populations studied in prior analyses [15]. More recent randomized trials for VAP prevention, though, provide insight into this important question. Put simply, VAP prevention trials allow one to inferentially assess the attributable mortality associated with VAP. More specifically, the attributable mortality of VAP is estimated to be approximately 10 percent and ranges between 3 and 17 percent [16]. A rigorously sound report attempted to address many of the limitations of prior analyses by employing a competing risk survival analysis. This allowed the authors to address the potential for either death while in the ICU or discharge from the ICU. Among 4479 patients in French ICUs, these investigators noted that VAP likely had a very low attributive mortality rate. Specially, they calculated that VAP increased the risk of death by 1 percent by day 30 and 1.5 percent by day 60 [17].

Regardless of mortality, VAP remains associated with significant costs. In a recent retrospective cohort study of 2238 patients, those with VAP had longer mean durations of MV. Mean hospitalization cost in one analysis, for instance, estimated that VAP nearly doubled over all hospital costs (to USD 99,598 in patients with VAP versus USD 59,770 for patients without VAP) [18]. Likewise, in a study of subjects following cardiac surgery in the United Kingdom, a diagnosis of VAP increased treatment costs by nearly GBP 9000 [19].

## 4. Diagnosis

Presently, the diagnosis of NP is based on clinical findings such as new onset of fever, leukopenia or leukocytosis, the presence purulent sputum, and new oxygen requirements. The diagnosis also necessitates the presence of new pulmonary infiltrates. Microbiological testing (see discussion below) is generally thought to be supportive of but not required for the diagnosis of VAP [20,21]. Unfortunately, this clinical paradigm has limited sensitivity and specificity. Although much has evolved in the NP literature relative to many topics, there have been few new works relating to diagnostics.

Despite the lack of relative new approaches for the diagnosis of NP, several studies have made clear the limitations of our current model. For example, Fernando et al. completed a meta-analysis of 25 reports addressing the diagnosis of NP. They estimated that the presence of infiltrates on a chest radiograph had a pooled sensitivity of 88.9% but a very low specificity of 26.1% [22]. A separate recent meta-analysis compared chest radiographs and lung ultrasounds (LUSs) for pneumonia diagnosis. Not surprisingly, chest radiographs had limited sensitivity and specificity. LUS, on the other, had an overall sensitivity of 95% and specificity of 94% [23]. These observations indicated that LUS has potential advantages over the radiograph as a screening tool for NP. Given the ease of use with LUS, this may represent a novel technology that comes to replace chest radiographs in coming years. 

Respiratory sampling and microbiological workups should be performed to identify the pathogens responsible for NP. Cultures of the lower respiratory tract, regardless of respiratory sampling methods, are currently the ‘gold-standard’ method in establishing the diagnosis of NP and confirming the causative organism. Conventional cultures require up to 24–36 h for incubation and identification of the possible pathogen and 48–72 h for antimicrobial susceptibility tests. The development of nonculture-based molecular diagnostics for common pathogens is responsible for nosocomial pneumonia, and their resistance genes from clinical samples have demonstrated improved detection time, therapy, and antibiotic stewardship. 

### Novel Diagnostics 

With respect to confirmatory microbiologic testing, all recognize the limitations of various culture techniques. Sputum cultures and tracheal aspirates, if they reveal growth, do not allow one to sort colonizing pathogens from potential infecting organisms. More invasive methods, including blind mini-brush or mini-bronchoalveolar lavage, are somewhat invasive, cannot be performed in non-intubated patients, and require quantitative colony counts. The contemporary INHALE WP1 study [24] compared the performance of two polymerase chain reaction (PCR) tests for VAP, the Unyvero and the BioFire FilmArray, in 652 respiratory samples collected from patients with suspected HAP/VAP at 15 ICU in the UK. In general, the PCR-based tests identified more pathogens compared to routine microbiology: 60.4% and 74.2% for Unyvero and FilmArray, respectively, vs. 44.2% by routine microbiology. For common pathogens, the FilmArray had a sensitivity of 91.7–100.0% and a specificity of 87.5–99.5%; Unyvero had a sensitivity of 50.0–100.0%, and specificity of 89.4–99.0%. The formal analysis indicated that, compared with PCR, routine microbiology suffered from low sensitivity, ranging from 27.0% to 69.4%. PCR; moreover, it afforded a more rapid identification of a culprit organism. Theoretically, one would expect PCR-based methods to allow more patients to receive initially appropriate antibiotic therapy more quickly.

A subsequent randomized trial enrolled 200 critically ill patients with pneumonia (115 with NP) [25]. The patients were assigned to either PCR testing using the FilmArray assay or to routine clinical care with traditional microbiologic testing. The proportion of patients receiving appropriate results-directed antibiotic therapy served as the primary endpoint. In the PCR group, 80% received results-directed therapy compared to only 30% of those in the control group. Additionally, 42% of subjects in the PCR group had antibiotics de-escalated compared with fewer than 10% of those in the control group. Consistent with the findings of the INHALE WP1 study, 71% of persons undergoing PCR testing had pathogens detected compared to 51% in the control arm. Just as LUS will likely become more routinely part of the approach to NP diagnosis, so will novel rapid diagnostic technologies.

## 5. Microbiology

### 5.1. Bacterial Pathogens

As noted earlier, a multitude of pathogens can lead to NP and include both traditional bacterial organisms and select viruses. For example, between 2015 and 2017, 9266 cases of VAP were reported to the United States Center for Disease Control and Prevention (CDC). Out of the 9266 cases, the most common Gram-positive bacteria included *Staphylococcus aureus* (28.8%), while the most common Gram-negative bacteria included *Pseudomonas aeruginosa* (12.9%) *Klebsiella* species (10.1%), and *Enterobacter* species (8.4%). In the US, *Acinetobacter baumanii* occurred rarely (3.2%) [26]. As alluded to above, the pathogens seen in nvHAP and vHAP are mostly similar to those that cause VAP. Zilberberg and co-workers [27], for example, reported that *S. aureus* represented the most common bacteria recovered in all forms of NP. *P. aeruginosa* and *Enterobacterales* were also seen commonly in nvHAP, vHAP, and VAP. *A. baumanii*, however, was twice as common in VAP as compared to either form of HAP. Despite these general similarities in pathogen type across distinct forms of NP, the prevalence of antimicrobial resistance appears to differ based on the type of NP. Carbapenem-resistance, for instance, was highest in VAP, while third-generation cephalosporins resistance was more prevalent in vHAP.

### 5.2. Viruses in Nosocomial Pneumonia 

Beyond bacteria, newer research underscores the potential significance of various respiratory viruses in NP. With recent enhancement in our ability to detect viruses as PCR-based approaches have improved, there has been a newer appreciation of the potential for the nosocomial transmission of viruses. In an earlier analysis by Shorr et al. [28], examining patients with ARDS, viruses alone were isolated in 21.7% of patients. Although most cases of viral infection arose in persons with community-onset infection, viral pathogens were seen in multiples cases of NP that progressed to respiratory failure. Strikingly, the crude hospital mortality rate associated with viral NP exceeded 35%. In contrast, in a more recent analysis (but one that still pre-dates the pandemic) focusing on VAP, viruses were only identified in 5.1% of subjects [29]. In this analysis, immunosuppression and stem-cell transplantation were independently associated with viral (as opposed to bacterial) pneumonia, but these variables performed poorly as screening tests for a viral infection. In short, clinicians need to recognize that viruses may lead to NP and that the prevalence of viral pathogens in various forms of NP appears to depend on the type of NP studied. 

## 6. COVID-19 and NP

One cannot discuss viruses and NP without addressing the impact of the pandemic on NP. The severe acute respiratory virus–coronavirus 19 (COVID-19) pandemic disrupted healthcare systems across the globe and had a major impact on intensive care-unit organization and operations. In terms of the pandemic’s impact on NP, many patients with COVID-19-related respiratory failure required prolonged MV. Thus, one might logically conclude that this would result in significantly higher rates of VAP and vHAP. The reported incidence of VAP during the pandemic, though, appeared to vary across multiple studies exploring this question. Fumagali et al. [30] reported that rates of VAP jumped dramatically and stated that nearly half of COVID-19 patients on MV developed VAP. Others described an attack rate for VAP ranging between 21 and 64%. Most of these reports come from analyses conducted in Europe. In the US, the rates of VAP were similar to those seen in Europe in more carefully conducted studies that relied upon lower-airway sampling. Pickens and colleagues determined that 44% of persons with COVID-19 on MV had microbiologic evidence of VAP, leading to an incidence of 44.5 cases per 1000 ventilator days [31]. In a systemic review by Jain et al., [32] the reported prevalence of VAP equaled 48% percent. Furthermore, in a retrospective cohort study comparing COVID-19 and influenza patients requiring ECMO, the cumulative incidence of VAP was significantly higher in COIVD-19 patients [33]. Why the higher incidence of VAP in COVID-19? There appears to be no one clear answer. Some have speculated that corticosteroid therapy for COVID-19 respiratory failure contributed to the high prevalence of VAP. Well-done analyses, though, suggest this is unlikely [34]. Readers should note that the bacterial pathogens responsible for VAP in COVID-19 do not appear to differ from those seen in VAP arising in other contexts. In a large systemic review of causative agents for VAP in COVID-19, the most common Gram-negative bacteria included *P. aeruginosa*, *K. pneumoniae*, *E. colacae*, and *A. baumannii*, just as one might see in traditional VAP [35].

## 7. Prevention

### 7.1. Oral Care

Multiple guidelines have previously addressed VAP prevention. However, in light of several important recent advances relating to VAP prevention, the Society for Healthcare Epidemiology (SHEA) and the IDSA updated their guidelines for VAP prevention in 2022 [36]. Although these recent revisions commented on several interventions and continued to stress the need for earlier extubation, head-of-the-bed elevation, and judicious use of stress ulcer prophylaxis, the main change to the guidelines was a strong recommendation for daily toothbrushing in MV patients. This recommendation was largely based on a well-done meta-analysis that evaluated 40 studies, which included 5675 patients [37]. Specifically, the authors of this report determined that oral care with toothbrushing (as opposed to placebo) reduced rates of VAP by nearly 30%. Though this review directly compared toothbrushing versus no toothbrushing, one should note that antiseptics, including chlorhexidine (CHG), were allowed in both control and intervention arms. This potentially represented an important confounder, particularly since CHG has not been recommended for clinical use since a large observational study observed higher death rates in persons exposed to CHG [38]. Since the publication of the SHEA/IDSA guideline, an additional meta-analysis relying on a different analytic paradigm reviewed seven trials and concluded that toothbrushing, in addition to chlorhexidine use, reduced the incidence of VAP by approximately a third as compared to merely chlorhexidine alone [39]. More strikingly, these investigators observed that routine toothbrushing reduced the duration of MV and ICU length of stay by approximately 1.5 days each. Building on these reports, Ehrenzeller and Klompas looked more broadly at over 15 studies of toothbrushing—including both ventilated and non-ventilated patients. Although they confirm the protective effects of toothbrushing in preventing NP, they, more importantly, estimate that toothbrushing not only reduced resource use but also reduced mortality in the ICU by approximately 20% [40]. Given the ubiquity of toothbrushing and the simplicity of the intervention, toothbrushing is now uniformly recommended by experts for VAP prevention. Prior confusion on this issue has been conclusively resolved. Further studies comparing toothbrushing alone versus toothbrushing in combination with CHG are warranted in order to better assess the potential value of CHG.

### 7.2. Pharmacologic Measures 

Beyond toothbrushing, several teams of researchers have explored pharmacologic means for VAP prevention. For example, in a well-designed multicenter, doubled-blind randomized control trial, Ehrmann and colleagues randomized MV patients to either inhaled amikacin at a dose of 20 mg/kg once daily or a placebo for 3 days [41]. Both interventions were delivered via an advanced jet nebulizer so as to enhance the likelihood that the drug reached the lower airways. Patients randomized to receive nebulized amikacin were significantly less likely to develop a first episode of VAP. More specifically, VAP occurred in 22% of placebo patients as compared to 15% of those randomized to inhaled amikacin. The authors further noted a delay in time to the diagnosis of VAP with inhaled amikacin.

Despite these positive findings, the significance of these results remains unclear. First, there was no reduction in the duration of MV with amikacin. If this intervention were meaningfully preventing a serious ICU complication, one would at least expect to see some difference in the overall duration of MV—as VAP certainly prolongs the duration of MV. Second, nebulized amikacin may simply be sterilizing upper-airway secretions and not truly preventing meaningful cases of VAP. Finally, if an intervention effectively prevented VAP, one would expect to see a lower need for antibiotic therapy among persons given the intervention. This, though, was not the case. Alternatively, the broad utilization of an aminoglycoside for disease prevention should give pause. It violates a general principle of antibiotic stewardship in that it may promote resistance. Though drug resistance is difficult to ascertain given the short duration of exposure in a single study, routine utilization of amikacin will undoubtedly promote resistance. Until further reports show clear morbidity or mortality benefits, nebulized amikacin for VAP prevention will likely not be routinely adopted or recommended.

Intravenous, as opposed to inhaled, administration of antibiotics has also recently been evaluated for VAP prevention. In the PROPHY-VAP trial, investigators randomized patients with brain injury to one dose of ceftriaxone or placebo for VAP prevention [42]. This multicenter, randomized trial was conducted in nine ICUs in France and included 319 patients. Enrolled patients were required to have a Glasgow Coma Score of 12 or less. Early VAP, defined as VAP occurring between 2 and 7 days of intubation, occurred in 14% of the treatment group versus 32% of the control group. In addition, it was found that ceftriaxone administration reduced the risk of being exposed to antibiotics and of eventually needing MV, and it decreased the ICU length of stay. Finally, mortality at day 28 was 15% in the group randomized to receive ceftriaxone versus 25% in the group that received placebo. Mechanistically, this paradigm’s mode of action appears intuitive. Acute brain injury, whether from trauma or stroke, represents a well-known risk factor for developing VAP, particularly early-onset VAP. Early-onset VAP, in such cases, is often caused by less virulent pathogens, such as *H. influenzae* and *S. aureus*. Additionally, in the brain-injured population, VAP likely arises either from aspiration at the time of insult or from endotracheal intubation. In this sense, ceftriaxone, in this population, can be viewed as akin to the use of a single dose of antibiotic prophylaxis prior to surgical incision in general surgery. Given the positive impact of this approach on key patient-centered outcomes, as well as the overall decrease in antibiotic utilization noted in the intervention group, a single dose of ceftriaxone for VAP prevention appears to represent a reasonable intervention in brain-injured patients. 

## 8. Treatment

Unfortunately, and despite one’s best efforts, attempts at preventing NP may fail. Hence, clinicians need to understand recent clinical trials addressing the treatment of NP, generally, and of VAP, specifically. The two major guidelines that exist for the management of NP are already outdated with respect to treatment, as they were initially released before 2018 [20,21]. Since the publication of these guidelines, several major trials of novel antibiotics have been published. These studies address several novel antibiotics and include trials of ceftolazone–tazbobactam, cefiderocol, imipenem–relebactam, and sulbactam–durlobactam.

### 8.1. Newer Antibiotics 

Carbapenem-resistant *Enterobacterales* (CRE) represents a growing concern both in the US and worldwide. Carbapenem resistance is estimated to occur in 2–7% of *Enterobacteriaceae* [43]. CRE has, more recently, become a relatively unhelpful concept, as this order of bacteria now exhibits multiple mechanisms of resistance. Generally, these CRE organisms can be divided into those that are carbapenemase-producing and those that do produce carbapenemases. Carbapenemase-producing isolates account for approximately 35–59% of CRE cases in the US, with the most common carbapenemase produced being the *K. pneumoniae* carbapenemase (KPC) [44]. Historically, the treatment of KPC-producing organisms relied upon polymyxin-based regimens, which have limited efficacy in pneumonia, and which expose the patient to significant toxicity [45]. Because of this, there has been a focused push to develop new antibiotics to treat CRE. Most recently, the RESTORE-IMI trials provided data for the utilization of the established carbapenem/renal dehydropeptidase inhibitor combination of imipenem–cilastatin, along with the novel beta lactamase relebactam [45,46]. The RESTORE-IMI 2 trial compared imipenem/cilastatin/relebactam to piperacillin–tazobactam for the treatment of HAP/VAP [46]. This double-blind, randomized controlled trial enrolled 537 patients from 113 hospitals. Imipenem/cilastatin/relebactam was found to be non-inferior to piperacillin–tazobactam for the treatment of NP based on all-cause 28-day mortality. In addition, in the subgroup of ventilated patients, the authors observed lower mortality in those randomized to imipenem/cilastatin/relebactam. In the RESTORE-IMI-1 trial, patients with difficult-to-treat pathogens were randomized to imipenem/cilastatin/relebactam or imipenem/cilastatin plus colistin [47]. Outcomes were similar in this small trial of very challenging-to-treat pathogens. However, nephrotoxicity occurred frequently with colistin. Based on the results of both studies, imipenem–cilastatin–relebactam now represents an acceptable option for KPC-producing organisms in pneumonia [43]. It is important to note that relebactam is the third novel β-lactamase inhibitor to be approved for use in the treatment of NP since 2018. Avibactam, combined with ceftazidime, was the first novel β-lactam inhibitor to be approved for the treatment of NP caused by CRE [48]. Shortly after, meropenem–vaborbactam became commercially available [49]. All three novel agents inhibit a wider range of β-lactamases, including Class A carbapenemases. Thus, they fill an important niche in medical practice [50]. It is important to note that these agents are not, however, interchangeable. For example, meropenem–vaborbactam does not have enhanced in vitro activity against multidrug-resistant *P. aeruginosa* [50]. Head-to-head studies directly comparing these agents are needed to help inform clinician decision-making. 

Class B and Class D carbapenemase-producing organisms represent an even more difficult-to-treat subset of CREs. Though their prevalence in the US remains low, they represent a major challenge in other parts of the globe [51]. It is important to note that none of the three antibiotics discussed above has activity against Class B carbapenemases. The CREDIBLE-CR study explored the efficacy and safety of cefiderocol, a novel siderophore cephalosporin that has in vitro activity against all four Ambler classes of β-lactamases, for the treatment of patients with infections caused by known, difficult-to-treat, carbapenem-resistant organisms [52]. In this small study, patients were randomized to either cefiderocol or the best available therapy. In patients with bloodstream infection, sepsis, or pneumonia, cefiderocol was permitted to be given with one adjunctive antibiotic, though 85% of patients randomized to cefiderocol received monotherapy. Though cefiderocol was found to have similar clinical and microbiological efficacy, it is important to note that 34% of the patients receiving cefiderocol died by the end of the study, whereas only 18% of the patients randomized to receive the best available therapy expired. This difference appeared to be driven mainly by excess deaths in the subset of patients with Acinetobacter infections. Regardless, cefiderocol was clearly efficacious against organisms producing Class B carbapenemases. An additional recent trial randomized patients with NP to either cefiderocol or high-dose meropenem. In this non-inferiority trial, cefiderocol was found to be non-inferior to meropenem [53].

*A. baumannii* represents an additional concerning carbapenem-resistant pathogen in NP [54,55]. Of note, carbapenem resistance among *A. baumannii* continues to rise at an alarming rate. Surveillance data reveal that carbapenem resistance more than doubled, from 21.0% in 2003 to 47.9% by 2012 [56]. A global analysis conducted between 2016 and 2018 found that resistance to meropenem was 67%, with the highest rates seen in Asia, Eastern Europe, and Latin America [54,56].

Prior to 2023, there were no available therapies proven to reduce mortality for this pathogen [54]. Hence, there was no preferred regimen for treating *A. baumannii* [44,56,57]. Recent guidelines for treating highly resistant pathogens developed by the European Society of Clinical Microbiology and Infectious Diseases (ESCMID) and by the IDSA both recommend combination therapy when treating moderate-to-severe carbapenem-resistant *A. baumanii* (CRAB) infections [44,57]. This suggestion is largely based on the probability that the use of two agents increases the likelihood that at least one agent has activity against CRAB. It is not based on any high-quality evidence suggesting a clinically meaningful synergistic benefit. Both guidelines recommend administering ampicillin–sulbactam in isolates that are susceptible, and the IDSA guidelines also suggest considering the use of ampicillin–sulbactam as part of combination therapy even in isolates that are not in vitro susceptible. A meta-analysis published in 2021 found that combination regimens including high-dose ampicillin–sulbactam were the most effective regimens in reducing mortality in patients with CRAB [58]. Unfortunately, this meta-analysis relied on few RCTs to support its conclusions. Although typically viewed simply as a β-lactamase inhibitor, sulbactam also exhibits direct antibacterial activity against *A. baumannii* through the saturation of essential penicillin binding proteins [44,55]. Other therapeutic options include minocycline, tigecycline, polymyxin B, colistin, extended-infusion meropenem, and cefiderocol. Given the significant toxicity associated with polymyxins, the need for better therapeutic options represents a key unmet clinical need. 

Durlobactam is a novel broad-spectrum diazabicyclooctane non-β-lactam β-lactamase inhibitor that is active against Ambler class A, C, and D β-lactamases. It has been shown to restore the activity of sulbactam against multidrug resistant *A baumannii* in in vitro studies [55]. A recently reported clinical trial assessed the efficacy and safety of sulbactam–durlobactam as compared to colistin in patients with CRAB infections [59]. The study relied on a novel rapid diagnostic to identify patients with the pathogen of interest so as to facilitate their rapid transition to in vitro active agents. In the end, sulbactam–durlobactam was found to be non-inferior to colistin and the vast majority of patients in this study suffered from NP. Although overall mortality was similar by day 28, more patients randomized to sulbactam–durlobactam were classified as clinical cures. Unsurprisingly, a significantly lower proportion of patients in the sulbactam–durlobactam group (12 (13%) out of 91) developed nephrotoxicity than in the colistin group (32 (38%) out of 85). 

Finally, it is important to recall the significance of multidrug-resistant *P. aeruginosa* in NP. As noted above, some novel beta-lactam/BLI combinations have activity against select multidrug-resistant strains of *P. aeruginosa*. Ceftolazone–tazobactam, however, is unique in that it provides an option for these *P. aeruginosa* infections but is narrower in its activity in that it does not cover CREs. Readers should recall that the approval of ceftolazone–tazobactam for use in NP was based on a randomized trial comparing this agent to meropenem. Unique in that it enrolled only ventilated patients, among those with vHAP, mortality was significantly lower among those treated with ceftolazone–tazobactam as opposed to meropenem [60]. A recent formal analysis of this pre-specified subgroup of subjects with vHAP reported that treatment with meropenem (as opposed to ceftolazone tazobactam) was independently associated with an increased risk of death [61].

Irrespective of the specific antibiotics employed for NP treatment, one key issue remains: shortening the patient’s exposure to antibiotics. In this sense, the findings of the just-published REGARD-VAP study validated the continued use of shorter antibiotic courses of therapy [62]. In this investigation, shorter courses of antibiotics for VAP proved to be non-inferior to longer treatment durations with respect to 60-day mortality and infection recurrence. In this trial, researchers randomized patients to individualized short-course treatment (based on clinical response) or usual care defined as less than 7 days (as short as 3–5 days) or greater than 8 days, respectively. Clinicians focused on fever resolution and hemodynamic stability to define clinical response. Median treatment duration in the short-course group was 6 days versus 14 days in the usual care group. This substantial reduction in antibiotic exposure was achieved, readers should note, without reliance on a formal biomarker.

### 8.2. Optimizing Pharmacokinetics 

Although newer agents will be crucial in the battle against MDR pathogens in NP, other potential means exist for optimizing antibiotic therapy. Recently, investigators have examined ways to leverage the pharmacokinetic properties of certain antibiotics. For beta-lactams, cephalosporins, and carbapenems, administering these agents via continuous infusion (rather than as a bolus) improves the time that the drug concentration is maintained above the minimum inhibitory concentration of the culprit pathogen. Exploiting this pharmacokinetic/pharmacodynamic attribute may lead to better outcomes and allow clinicians to treat MDR organisms with agents to which a pathogen might otherwise be non-susceptible. Unfortunately, the most recent large, randomized trial of this strategy failed to affect patient outcomes. Specifically, Monti and colleagues in a multicenter study randomized over 600 patients with sepsis or septic shock to either continuous infusion or bolus administration of meropenem [63]. Approximately a third of the subjects had a respiratory infection. Continuous infusion was not associated with either an improvement in mortality or the prevention of AMR on therapy [63]. In short, further studies will be needed to determine if continuous antibiotic infusion improves important outcomes and, if it does, what types of patients are most likely to benefit. 

## 9. Conclusions

NP will certainly remain a major challenge for both patients and clinicians. As patients in the hospital remain more severely ill than in the past, or as they undergo ever more complex major surgeries, the threat of NP will remain. This concern, as the pandemic reminded us, remains particularly true for those who eventually require prolonged courses of MV. Moreover, the threat of AMR is likely to worsen in the near term before it improves—if it ever does. Over the last several years, however, multiple key analyses have explored central issues related to NP. These reports have demonstrated that not all HAP patients face similar risks, while also underscoring the economic and morbidity penalties associated with NP. Although newer diagnostic approaches appear to be on the horizon for NP, prevention remains key to improving outcomes. Fortuitously, simple interventions such as daily oral care can have a major positive impact. Several recent randomized studies in the area of NP therapeutics demonstrate the efficacy of multiple novel agents designed to address many of the most problematic pathogens. It remains incumbent on clinicians to appreciate the significance of these recent studies and to determine how best to apply their lessons at the bedside. It is only through a measured yet rigorous application of the best evidence that we can hope to improve outcomes for our patients suffering from NP.

## Data Availability

Not Applicable.
